# Recurrent Mutations in *BRCA1*, *BRCA2*, *RAD51C*, *PALB2* and *CHEK2* in Polish Patients with Ovarian Cancer

**DOI:** 10.3390/cancers13040849

**Published:** 2021-02-18

**Authors:** Alicja Łukomska, Janusz Menkiszak, Jacek Gronwald, Joanna Tomiczek-Szwiec, Marek Szwiec, Marek Jasiówka, Paweł Blecharz, Tomasz Kluz, Małgorzata Stawicka-Niełacna, Radosław Mądry, Katarzyna Białkowska, Karolina Prajzendanc, Wojciech Kluźniak, Cezary Cybulski, Tadeusz Dębniak, Tomasz Huzarski, Aleksandra Tołoczko-Grabarek, Tomasz Byrski, Piotr Baszuk, Steven A. Narod, Jan Lubiński, Anna Jakubowska

**Affiliations:** 1 Department of Genetics and Pathology, Pomeranian Medical University in Szczecin, 71-252 Szczecin, Poland; alicja.lukomska@pum.edu.pl (A.Ł.); jgron@pum.edu.pl (J.G.); katarzyna.bialkowska@pum.edu.pl (K.B.); karolina.prajzendanc@pum.edu.pl (K.P.); kluzniak@pum.edu.pl (W.K.); cezarycy@pum.edu.pl (C.C.); debniak@pum.edu.pl (T.D.); huzarski@pum.edu.pl (T.H.); otjg@interia.pl (A.T.-G.); piotr.baszuk@pum.edu.pl (P.B.); lubinski@pum.edu.pl (J.L.); 2 Department of Gynecological Surgery and Gynecological Oncology of Adults and Adolescents, Pomeranian Medical University in Szczecin, 70-111 Szczecin, Poland; nbz@list.pl; 3 Department of Histology, Department of Biology and Genetics, Faculty of Medicine, University of Opole, 45-040 Opole, Poland; jszwiec@uni.opole.pl; 4 Department of Surgery and Oncology, University of Zielona Góra, 65-046 Zielona Góra, Poland; m.szwiec@wln.uz.zgora.pl; 5 Department of Chemotherapy Ludwik Rydygier Memorial Specialist Hospital, Zlotej Jesieni 1, 31-820 Krakow, Poland; marek.jasiowka@onkologia.krakow.pl; 6 Department of Gynaecologic Oncology, Maria Sklodowska-Curie National Research Institute of Oncology, Krakow Branch, 31-115 Krakow, Poland; pawel.blecharz@interia.pl; 7 Department of Gynecology and Obstetrics, Institute of Medical, Sciences, Medical College of Rzeszow University, 35-959 Rzeszow, Poland; jtkluz@interia.pl; 8 Department of Clinical Genetics and Pathology University of Zielona Góra, 65-046 Zielona Góra, Poland; m.stawicka-nielacna@wlnz.uz.zgora.pl; 9 Department of Gynecological Oncology, Poznan University of Medical Science, 60-569 Poznań, Poland; radoslaw.madry@skpp.edu.pl; 10 Department of Oncology and Chemotherapy, Pomeranian Medical University in Szczecin, 70-111 Szczecin, Poland; tomasz.byrski@pum.edu.pl; 11 Women’s College Research Institute, Toronto, ON M5S 1B2, Canada; Steven.Narod@wchospital.ca; 12 Independent Laboratory of Molecular Biology and Genetic Diagnostics, Pomeranian Medical University in Szczecin, 71-252 Szczecin, Poland

**Keywords:** ovarian cancer, recurrent mutations, *BRCA1*, *BRCA2*, *RAD51C*, *PALB2*, *CHEK2*, cancer risk

## Abstract

**Simple Summary:**

In Poland, ovarian cancer is the fourth leading cause of death from cancer among women. Several founder mutations in the *BRCA1*, *BRCA2*, *PALB2*, *RAD51C*, and *CHEK2* genes are associated with breast and ovarian cancer. The aim of the study was to analyze the frequency and magnitude of association of 21 recurrent founder germline mutations in the above genes with ovarian cancer risk among unselected patients in Poland. The ovarian cancer risk was associated with mutations in *BRCA1*, *BRCA2*, *RAD51C*, and *PALB2* but not in the *CHEK2* gene. Excluding *CHEK2*, pathogenic mutations in the other 18 alleles were present in 12.5% of cases and 0.6% of healthy controls. A mutation was found in 25.8% of familial cases vs. 9.9% of non-familial cases. We recommend that in Poland all women with ovarian cancer and first-degree female relatives should be tested for the panel of 18 founder mutations in *BRCA1*, *BRCA2*, *PALB2*, and *RAD51C*.

**Abstract:**

The aim of the study was to analyze the frequency and magnitude of association of 21 recurrent founder germline mutations in *BRCA1*, *BRCA2*, *PALB2*, *RAD51C*, and *CHEK2* genes with ovarian cancer risk among unselected patients in Poland. We genotyped 21 recurrent germline mutations in *BRCA1* (9 mutations), *BRCA2* (4 mutations), *RAD51C* (3 mutations), *PALB2* (2 mutations), and *CHEK2* (3 mutations) among 2270 Polish ovarian cancer patients and 1743 healthy controls, and assessed the odds ratios (OR) for developing ovarian cancer for each gene. Mutations were detected in 369 out of 2095 (17.6%) unselected ovarian cancer cases and 117 out of 1743 (6.7%) unaffected controls. The ovarian cancer risk was associated with mutations in *BRCA1* (OR = 40.79, 95% CI: 18.67–114.78; *p* = 0.29 × 10^−15^), in *BRCA2* (OR = 25.98; 95% CI: 1.55–434.8; *p* = 0.001), in *RAD51C* (OR = 6.28; 95% CI 1.77–39.9; *p* = 0.02), and in *PALB2* (OR 3.34; 95% CI: 1.06–14.68; *p* = 0.06). There was no association found for *CHEK2.* We found that pathogenic mutations in *BRCA1*, *BRCA2*, *RAD51C* or *PALB2* are responsible for 12.5% of unselected cases of ovarian cancer. We recommend that all women with ovarian cancer in Poland and first-degree female relatives should be tested for this panel of 18 mutations.

## 1. Introduction

In Poland, ovarian cancer is the second most common gynecological cancer and the fourth leading cause of death from cancer among women, with 1.5% as the average lifetime risk for developing ovarian cancer [1,2]. In 2017, there were 3775 new cases diagnosed and 2670 reported deaths [3]. Several factors have been shown to reduce the risk of ovarian cancer, including oral contraceptive use [4], especially observed in the last few decades [5], high parity [6], and breast-feeding [7]. Ovarian cancer often presents at a late stage (70% in FIGO stage III or IV) [8] due to the lack of effective screening programs and because early-stage disease is typically asymptomatic [9].

A family history of ovarian cancer is a strong risk factor and several predisposing genes have been identified [10]. Approximately one-fifth of ovarian cancers have been associated with a mutation in one of these genes. The most important genetic causes of ovarian cancer are mutations in *BRCA1* and *BRCA2* genes, which account for up to 15% of all cases [11,12,13,14]. *BRCA1* and *BRCA2* encode proteins involved in recombinational repair of damaged DNA [11] and it has been found that mutations in other genes involved in the repair of DNA damage by homologous recombination, including e.g., *RAD51C*, *RAD51D*, *BRIP1*, *PALB2*, and *CHEK2* may be associated with ovarian cancer risk [15,16,17,18,19,20,21].

In Poland, founder mutations in *BRCA1*, *BRCA2*, *PALB2*, *CHEK2*, and *RAD51C* have been associated with familial breast and ovarian cancer. Founder mutations in *BRCA1* (nine alleles) and *BRCA2* (four alleles) are frequent among Polish breast and ovarian cancer patients [12,13,14,22,23,24]. The other known founder mutations were identified in *PALB2* (two alleles), *CHEK2* (four allele) and recently *RAD51C* (three alleles, data not published) genes [15,18,21,25,26,27]. It has been shown that population-based genetic testing in defined populations, independent of personal or family history of cancer may be a useful approach for determining cancer susceptibility [28]. Founder mutations can be detected rapidly and at relatively low cost in specific ethnic-groups. Until now in Poland, this approach has been applied for testing of founder *BRCA1/2*, *PALB2*, and *CHEK2* mutations in association with breast cancer risk [23,24,29,30]. The prevalence and association of the founder mutations with ovarian cancer risk in the Polish population was limited to analysis of *BRCA1/2* in relatively small groups of cases [12,13,14,25,31], and was not performed for other genes.

The aim of the study was to analyze the prevalence and association of recurrent founder germline mutations in *BRCA1*, *BRCA2*, *RAD51C*, *PALB2*, and *CHEK2* genes with ovarian cancer risk among unselected patients in the Polish population. In addition, we estimated the hazard ratio for all-cause survival among mutation carriers in comparison to non-carriers.

## 2. Results

### 2.1. Association of BRCA1, BRCA2, RAD51C, PALB2, and CHEK2 Mutations with Ovarian Cancer Risk

Genotyping of 21 mutations in *BRCA1*, *BRCA2*, *RAD51C*, *PALB2*, and *CHEK2* genes was performed for all 2270 ovarian cancer patients and 1743 healthy controls. For 175 ovarian cancer patients, genotyping was incomplete and therefore these cases were excluded, leaving finally 2095 ovarian cancer patients in further analyses.

The highest frequency of tested mutations in ovarian cancer patients was detected for the *BRCA1* gene. One of 9 mutations in *BRCA1* was detected in 220 out of 2095 (10.5%) ovarian cancer cases and in 5 out of 1743 controls (0.29%), and was associated with odds ratio 40.8 (95% CI: 18.7–114.8; *p* = 0.29 × 10^−15^). One of 4 tested *BRCA2* mutations was found in 15 out of 2095 (0.72%) unselected ovarian cancer patients but in none of 1743 controls, and was associated with odds ratio 26.0 (95% CI: 1.55–434.8; *p* = 0.001).

One of 3 mutations in *RAD51C* was seen in 15 out of 2095 (0.72%) ovarian cancer cases and in 2 out of 1743 controls (0.11%) (OR = 6.28; 95% CI 1.77–39.9; *p* = 0.02).

One of 2 mutations in *PALB2* was detected in 12 out of 2095 (0.57%) cases and in 3 of 1743 controls (0.17%) (OR = 3.34; 95% CI 1.06–14.68; *p* = 0.06).

The three mutations in the *CHEK2* gene were analyzed separately with respect to the consequences for proteins. Two protein truncating mutations (c.1100delC, c.444+1G>A) were detected in 17 cases (0.81%) and 10 controls (0.57%). The missense mutation (c.470T>C) was found in 90 cases (4.3%) and 97 controls (5.57%). Neither of these differences was statistically significant.

The distributions of the tested mutations in *BRCA1*, *BRCA2*, *PALB2*, and *RAD51C* genes and odds ratios (ORs) for risk of ovarian cancer are shown in Table 1.

Excluding *CHEK2*, a pathogenic mutation was present in 262 of 2095 cases of ovarian cancer (12.5%). A mutation was found in 25.8% of familial cases and in 9.9% of non-familial cases. The mutations were detected in 103 (39.3%) of women diagnosed under age 50, in 102 (38.9%) of women diagnosed between age 50 and 60, and in 57 (21.8%) of women diagnosed above the age of 60. In regard to the histopathology of ovarian cancer, the mutations were found in 14.0% of serous cases, 5.2% of endometrioid cases, 3.6% of mucinous, 1.6% of clear cell cases, and 6.0% of cases with other or unspecified histology.

An analysis of the presence of mutations in *BRCA1*, *BRCA2*, *RAD51C*, *PALB2*, and *CHEK2* with the age of onset of ovarian cancer revealed a lower age of ovarian cancer diagnosis in *BRCA1* mutation carriers than in non-carriers of *BRCA1* (51.6 vs. 57.6, *p* = 2.97 × 10^−11^). This was not found in carriers of mutations in *BRCA2*, *RAD51C*, *PALB2* or *CHEK2.* There was no statistically significant association of mutations with age of onset among patients with a positive family history of ovarian cancer.

### 2.2. All-Cause Survival

Information on all-cause survival was available from 1924 cases and 1084 deaths were recorded (56.3%). The 5-year survival for all patients was 47.2% and the 10-year survival was 26.1%. The 5- and 10-year survival of mutation carriers, except of *CHEK2* protein truncating, was in general longer than for non-carriers (Table 2). Among carriers of *CHEK2* protein truncating mutations, 50% of patients survived 5 years from ovarian cancer diagnosis, but all died within 10 years.

The estimated hazard ratio for all-cause survival among mutation carriers was not statistically significant for any of the tested genes (Table 3).

## 3. Discussion

The goal of our study was to estimate the prevalence of 21 founder/recurrent germline mutations among unselected ovarian cancer patients in Poland, and to assess the odds ratios (ORs) for developing ovarian cancer. Mutations were detected in 369 out of 2095 (17.6%) unselected ovarian cancer cases and 117 out of 1743 (6.7%) unaffected controls.

*BRCA1* mutations were much more frequent than *BRCA2* mutations (10.5% versus 0.72%, respectively). These mutations were associated with odds ratios of 40.79 (95% CI: 18.67–114.78; *p* = 0.29 × 10^−15^) and 25.98 (95% CI: 1.55–434.8; *p* = 0.001), for *BRCA1* and *BRCA2*, respectively. Previous studies of Polish ovarian cancer patients were much smaller, ranging from 134 to 364 cases [12,13,14,25,31]. Full sequencing of all exons of *BRCA1* and *BRCA2* suggests that the founder/recurrent mutations tested in the current study constitute about 65% of all mutations in Poland [14,25]. The results of our study are concordant with previously reported analyses which show a high prevalence of *BRCA1/2* mutations among ovarian cancer patients with dominant founder/recurrent population-specific mutations located in *BRCA1*.

Mutations in *RAD51C*, *PALB2*, and *CHEK2* genes were previously analyzed in ovarian cancers [15,19,21,26]. In our study we show and confirm that in Poland mutations in *RAD51C* and *PALB2* are associated with ovarian cancer risk.

Mutations in the *RAD51C* gene were reported to predispose to ovarian cancer, as well as to breast cancer but only in families with ovarian cancer cases [15,16,17,25,32,33]. In the meta-analysis encompassing approximately 24,000 ovarian cancer patients from 53 studies and more than 100,000 controls, mutations in *RAD51C* were associated with a 5-fold elevated risk of ovarian cancer (OR = 5.59; 95%CI: 4.42–7.07; *p* < 0.0001) [16]. In our study one of three tested recurrent *RAD51C* germline mutations was detected in 15 out of 2095 (0.72%) unselected ovarian cancer patients giving the odds ratio of 6.28 (95% CI: 1.77–39.87; *p* = 0.02).

Germline mutations in *PALB2* were reported to correlate with an increased risk of ovarian cancer [18,19,34,35]. In Poland there is little data about association of *PALB2* mutations with ovarian cancer. In 2 studies of 333 unselected and 339 unrelated ovarian cancer cases, the *PALB2* mutations were detected in 0.6% of patients [25,26]. In our analysis recurrent *PALB2* mutations were detected in 12 out of 2095 (0.57%) unselected ovarian cancer cases giving the odds ratio of 3.34, but the difference was of borderline statistical significance (95% CI: 1.06–14.6; *p* = 0.06). This is concordant with results of analyses performed on other populations which showed 3–4 times higher frequency of *PALB2* mutations in ovarian cancer patients than in controls [18,19,34,35]. Comparison of prevalence of *PALB2* mutations between studies suggests that recurrent mutations are more common in Poland than in other populations.

*CHEK2* mutations are known to increase the risk of prostate and breast cancer [29,36,37,38]. With regards to ovarian cancer, the results are inconsistent [21,25,27,37,39]. In our study there was no significant association of tested *CHEK2* mutations, including 2 protein truncation and 1 missense mutation, with ovarian cancer risk.

In our analysis one of the *BRCA1*, *BRCA2*, *RAD51C*, and *PALB2* mutations was found in 25.8% of familial cancer cases and in 9.9% of non-familial cases, similar to what has been reported previously [18,33,40,41].

We did not have access to specific causes of death and we estimated the hazard ratios for all-cause survival among carriers of mutations in *BRCA1*, *BRCA2*, *RAD51C*, *PALB2*, and *CHEK2* genes in comparison with women who carried no mutation. There was no significant association of mutation status in any of the tested genes with overall all-cause survival among carriers at 5 or 10 years. Several studies, including a large meta-analysis encompassing almost 8000 ovarian cancer cases, have reported that *BRCA1* and *BRCA2* carriers have better survival than non-carriers [13,42,43,44,45,46], but the survival advantage diminishes with time from diagnosis.

We show significant correlation of mutations in *BRCA1*, *BRCA2*, and *RAD51C* with ovarian cancer risk. The association was also detected for *PALB2* mutations, but it was of borderline statistical significance. It will require analyses on larger sample sizes to provide precise estimates for *PALB2* for clinical counselling [18,19,34].

The results of this study based on analysis of selected founder mutations in ovarian cancer patients may have important implications for the population. Panel testing offers a cheaper alternative to methods based on whole gene DNA-sequencing and therefore may be applicable to testing of the female population at large. Testing women for the panel of population-specific recurrent/founder mutations may be a valuable approach for therapy decisions in ovarian cancer patients (i.e., platinum or PARP inhibitors in *BRCA1/2* carriers) or for preventing hereditary ovarian cancer. Women who are found to carry a mutation in one of these genes would be candidates for preventive salpingo-oophorectomy which has been shown to be an effective means of cancer control.

## 4. Materials and Methods

### 4.1. Study Groups

The study group consisted of 2270 unselected ovarian cancer patients diagnosed between 1998 and 2019 at the International Hereditary Cancer Center (IHCC) in Szczecin and elsewhere in Poland (Kraków, Rzeszów, Opole, Zielona Góra, Poznań). The clinical information included age at diagnosis, tumor histology, and family history of breast and ovarian cancers. Data on vital status were collected from the Ministry of Digital Affairs in July 2019. The characteristics of ovarian cancer patients are presented in Table 4.

The control group consisted of 1743 healthy women selected from the registry of the International Hereditary Cancer Center in Szczecin. They were recruited between 2002–2019 as a part of the population-based study of the approximately 2 million inhabitants of the West Pomerania region in Poland, designed to identify familial aggregations of cancer syndromes, or they independently came to the International Hereditary Cancer Center for genetic counseling. For all individuals we collected demographic and clinical data as well as information about family history of cancer. The mean age of controls at the time of recruitment was 55.8 years (range 18–90 years).

Healthy controls were followed since time of recruitment, and the mean follow up was 26.1 months (0–213 months).

### 4.2. Sample Preparation and Molecular Analyses

A blood sample was obtained from all cases and controls and DNA was isolated using a previously described method [47]. DNA samples were stored at 4 °C prior to genotyping. The analysis encompassed genotyping of 21 selected recurrent/founder germline mutations localized in five genes: *BRCA1* (9 mutations), *BRCA2* (4 mutations), *RAD51C* (3 mutations), *PALB2* (2 mutations), and *CHEK2* (3 mutations) (Table 5).

All mutations, with the exception of c.5266dupC in *BRCA1*, were genotyped by Real-Time PCR using customized TaqMan Assays on LightCycler Real-Time PCR 480 System (Real-Time PCR System, Roche Diagnostics, Indianapolis, IN, USA) following the standard protocol. The reaction mix for analysis of each sample included GoTaq^®^ Probe qPCR Master Mix (Promega, Madison, WI, USA), TaqMan Genotyping Assays × 40 or TaqMan Genotyping Assays × 80 (Applied Biosystems, Foster City, CA, USA), and deionized water (Promega, Madison, WI, USA). Samples were analyzed on 384-well plates. On each analyzed plate were included 3 control samples: positive and negative for tested mutations, and a water-blind control. The genotyping was performed using LightCycler 480 Instrument and data were analyzed using LightCycler 480 Basic Software Version 1.5 (Roche Diagnostics, Indianapolis, IN, USA). Analysis for c.5266dupC mutation in *BRCA1* was performed using allele-specific amplification PCR (ASA-PCR) as described previously [33]. The sequences of primers and probes used for genotyping are available on request.

All study participants provided written informed consent. The study was approved by the Ethics Committee of the Pomeranian Medical University in Szczecin.

### 4.3. Statistical Analysis

To analyze the association of tested mutation with ovarian cancer risk, odds ratios were estimated separately for each gene, *BRCA1*, *BRCA2*, *RAD51C*, *PALB2*, and *CHEK2*. The odds ratios (ORs) and corresponding 95% confidence intervals (95% CIs) were calculated using a logistic regression model.

In addition, the Mann–Whitney or T-test was used for comparison of the age of ovarian cancer onset between patients with and without mutation. This analysis was performed separately in patients with positive and negative family history of ovarian cancer.

To estimate overall survival, all ovarian cancer patients were followed from the date of diagnosis until death from any cause. Analysis was performed for each gene separately (*BRCA1*, *BRCA2*, *RAD51C*, *PALB2*, and *CHEK2*) using Cox proportional hazards. In addition, the 5- and 10-year survival among the *BRCA1*, *BRCA2*, *RAD51C*, *PALB2*, and *CHEK2* mutation carriers was calculated.

All analyses were done in R Project for Statistical Computing (version 3.5.2) and *p*-values less than 0.05 were recognized as statistically significant.

## 5. Conclusions

We found that a pathogenic mutation in *BRCA1*, *BRCA2*, *RAD51C* or *PALB2* was found in 12.51% of unselected cases of ovarian cancer in Poland. We recommend that all women with ovarian cancer in Poland and first-degree female relatives be tested for this panel of 18 mutations.

## Figures and Tables

**Table 1:** The prevalence and association of tested 21 founder/recurrent mutations in *BRCA1*, *BRCA2*, *RAD51C*, *PALB2*, and *CHEK2* genes with ovarian cancer risk.

Mutation	Cases, n = 2095 (%)	Controls, n = 1743 (%)
*BRCA1*		
5382insC (c.5266dupC)	124 (5.92)	3 (0.17)
300T>G (c.181T>G)	48 (2.29)	1 (0.06)
4153delA (c.4035delA)	10 (0.48)	0 (0.00)
1806C>T (c.1687C>T)	6 (0.29)	0 (0.00)
185delAG (c.68_69delAG)	8 (0.38)	0 (0.00)
3819del5 (c.3700_3704del5)	14 (0.69)	1 (0.06)
3875del4 (c.3756_3759delGTCT)	3 (0.14)	0 (0.00)
5370C>T (c.5251C>T)	1 (0.05)	0 (0.00)
794delT (c.794_795delCT)	6 (0.29)	0 (0.00)
Any *BRCA1* mutation	220 (10.50)	5 (0.29)
OR 40.79 (95% CI: 18.67–114.78), *p* = 0.29 × 10^−15^		
*BRCA2*		
4075delGT (c.3847_3848delGT)	3 (0.14)	0 (0.00)
8138del5 (c.7913_7917delTTCCT)	3 (0.14)	0 (0.00)
886delGT (c.658_659del)	4 (0.19)	0 (0.00)
6174delT (c.5946delT)	5 (0.24)	0 (0.00)
Any *BRCA2* mutation	15 (0.72)	0 (0.00)
OR 25.98 (95% CI: 1.55–434.8), *p* = 0.001		
*RAD51C*		
c.905-2_905-1delAG	2 (0.10)	2 (0.11)
c.577C>T	10 (0.48)	0 (0.00)
c.502A>T	3 (0.14)	0 (0.00)
Any *RAD51C* mutation	15 (0.72)	2 (0.11)
OR 6.28 (95% CI: 1.77–39.87), *p* = 0.02		
*PALB2*		
c.172_175delTTGT	4 (0.19)	2 (0.11)
c.509_510delGA	8 (0.38)	1 (0.06)
Any *PALB2* mutation	12 (0.57)	3 (0.17)
OR 3.34 (95% CI: 1.06–14.68), *p* = 0.06		
*CHEK2*		
c.1100delC	6 (0.29)	4 (0.23)
c.444+1G>A	11 (0.53)	6 (0.34)
Both protein truncating *CHEK2* mutation	17 (0.81)	10 (0.57)
OR 1.42 (95% CI: 0.66–3.22), *p* = 0.38		
missense mutation, c.470 T>C	90 (4.30)	97 (5.57)
OR 0.76 (95% CI: 0.57–1.02), *p* = 0.07		

**Table 2:** The 5- and 10-year survival of ovarian cancer patients by mutation status.

Survival	5-Year Survival Rate (%)	10-Year Survival Rate (%)
*BRCA1*	50.53%	27.04%
*BRCA2*	54.55%	40.00%
*RAD51C*	58.33%	40.00%
*PALB2*	54.55%	33.33%
*CHEK2*, c.1100delC and c.444+1G>A	50.00%	- *
*CHEK2*, c.470T>C	54.17%	34.48%
Non-carriers	46.82%	25.71%

* All carriers died.

**Table 3:** Association of *BRCA1*, *BRCA2*, *RAD51C*, *PALB2*, and *CHEK2* mutations with all-case survival among unselected ovarian cancer patients.

Mutation	Hazard Ratio (95% CI)	*p*-Value
*BRCA1*	0.98 (0.81–1.18)	0.81
*BRCA2*	0.87 (0.43–1.75)	0.70
*RAD51C*	0.63 (0.28–1.41)	0.26
*PALB2*	0.89 (0.42–1.88)	0.76
*CHEK2*, c.1100delC and c.444+1G>A	1.14 (0.59–2.20)	0.69
*CHEK2*, c.470T>C	0.80 (0.58–1.10)	0.17

**Table 4:** Characteristics of ovarian cancer patients.

Feature	n = 2270
Age of diagnosis (mean, range)	57 (19–87)
Histological type of cancer	
Serous	1133 (49.9%)
Clear-cell	70 (3.1%)
Endometrioid	286 (12.6%)
Mucinous	147 (6.5%)
Other or undefined/mixed	38 (1.7%)
Unknown	596 (26.3%)
Family history of ovarian cancer	
Yes	334 (14.7%)
No	1633 (71.9%)
Unknown	303 (13.3%)
Family history of BC	
Yes	372 (16.4%)
No	1615 (71.1%)
Unknown	283 (12.5%)
Mean follow up in years (range)	10.1 (0.1–21.0)
Death (all causes)	
Yes	1179 (51.9%)
No	895 (39.4%)
Unknown	196 (8.6%)

**Table 5:** Recurrent mutations tested in *BRCA1*, *BRCA2*, *RAD51C*, *PALB2*, and *CHEK2* genes.

Gene	cDNA	Protein Change	Molecular Consequence
*BRCA1*	5382insC (c.5266dupC)	p.Gln1756fs	frameshift
	300T>G (c.181T>G)	p.Cys61Gly	missense
	4153delA (c.4035delA)	p.Glu1346fs	frameshift
	1806C>T (c.1687C>T)	p.Gln563Ter	nonsense
	185delAG (c.68_69delAG)	p.Glu23fs	frameshift
	3819del5 (c.3700_3704del5)	p.Val1234fs	frameshift
	3875del4 (c.3756_3759delGTCT)	p.Ser1253fs	frameshift
	5370C>T (c.5251C>T)	p.Arg1751Ter	nonsense
	794delT (c.794_795delCT)	p.Ser265Cysfs	frameshift
*BRCA2*	4075delGT (c.3847_3848delGT)	p.Val1283fs	frameshift
	8138del5 (c.7913_7917delTTCCT)	p.Ala2637_Phe2638insTer	nonsense
	886delGT (c.658_659del)	p.Val220fs	frameshift
	6174delT (c.5946delT)	p.Ser1982fs	frameshift
*RAD51C*	c.905-2_905-1delAG	p.Glu303TrpfsX41	skipping of exon 7
	c.577C>T	p.Arg193Ter	nonsense
	c.502A>T	p.Arg168Ter	nonsense
*PALB2*	c.172_175delTTGT	p.Gln60fs	frameshift
	c.509_510delGA	p.Arg170fs	frameshift
*CHEK2*	c.1100delC	p.Thr367fs	frameshift
	c.444+1G>A	p.E149IfsX6	skipping of exon 2
	c.470T>C	p.Ile157Thr	missense

## Data Availability

The data presented in this study are available on request from the corresponding author.
